# Supplementary material for: Patterns of Intron Gain and Loss in Fungi
Source: PLoS Biol. 2004 Nov 30;2(12):e422. doi: 10.1371/journal.pbio.0020422 (PMC532390; doi:10.1371/journal.pbio.0020422)
Supplement: Table S1 — Also available at http://genes.mit.edu/NielsenEtAl/. (4.3 MB ZIP). [file pbio.0020422.st001.zip › NielsenEtAl/html/286.html]

AN5635.1.NCU04221.1.MG09471.1.FG09895.1


```
 CLUSTAL W (1.82) Multiple Sequence Alignments - Introns Inserted


Sequence 1: NCU04221.1	744 aa
Sequence 2: MG09471.1	736 aa
Sequence 3: FG09895.1	738 aa
Sequence 4: AN5635.1	748 aa
Alignment Length: 754 aa
Number Identitical Residues: 415 aa
Alignment Score (without introns) 20614


MG09471.1 	--MSEAPQARRVG---------SVDDHSVYDDAKTYYTSEERHNNSRSGPRQRTY~SQ0N
NCU04221.1	--MTSQPSSGKGRGR-----NLSIDEYNVYDDAKTYYTTEDRHHNHRAGARTRTY~SQ0N
FG09895.1 	---MAAPSNHRSR---------GSDDLGVFDDAKSYYT-AERHQN-RAGPRTRTY~SQ0N
AN5635.1  	MDDSALPSNTSNGINGRTAHRRSSSGGDPFQHPDIYYGNPESVERIKNRRRAFSS0SL~K
          	 .    *.       . ::   . .  . ::... ** . :  .. :   *  :  *  :

MG09471.1 	SLLGQMERLGL-KEPFRRGSHD1ES--NHNRRFLIQVDPTLESLKSQEDTDGNMQITIED
NCU04221.1	SLFKQFERLGLQKEPYRRGSHD1ESTIPQSRRFLIQVEPTLQSLQSQEDTDGNMQITIED
FG09895.1 	SLMSRFERVNL-REPFRRGSHD1ENS-QQNRRFLIQVDSTLESLSLQEDPNGNMQITIED
AN5635.1  	SFNRQDFHEMLGDRNTRRGSMD1PTS-GNPRKFLIDVDATLHSLLEREDSDRNMQITIED
          	*:  :  :  *  .  **** *  .:  : *:***:*:.**.**  :**.: ********

MG09471.1 	NGPK0VL~TLRTAGSNGHNRFDIRGTYMLSNLLQELTLAQEYGRKQVILDEARLNENPVN
NCU04221.1	NGPK0VL~SLRTAASNGYNRFDIRGTYMLSNLLQELYLAKEYGRKQIILDEARLNENPVN
FG09895.1 	NGPK~VI~SLRTAASAGHNRFDVRGTYMLSNLLQELTLAKEYGRKQIVLDEGRLNENPVD
AN5635.1  	VGPK~VR1---TAASHGYNRFDVRGTYMLSNLLQELTIAKDYGRKQIVLDEERLSENPVS
          	 *** *     **.* *:****:************* :*::*****::*** **.****.

MG09471.1 	RLSRLIRDHFWDALTRRIDASSIEVAAKDPKDWTDDPRPRIYVPKGAPEQLEYYKKLAAD
NCU04221.1	RLSRLIKDHFWEGLTRRIDASSIEIAARDPKDWTDDPRPRIYIPRGAPEQHEYYTKVALD
FG09895.1 	RLSRMIRDHFWENLTRRIDASTVDIAARDPKDWTADPRPRIYIPYRCPRQYEFYKRVAEE
AN5635.1  	RLSRLIKNSFWNSLTRRIDGRNIEVAGRDPKDWTDDPRPRIYVPPGAPEQLEYYRRIAEE
          	****:*:: **: ******. .:::*.:****** *******:*  .*.* *:* ::* :

MG09471.1 	KPDIRLDVVELPETITPEYVVGINKAPGLLAVDMEETVDP-KTGERVMSGRPFVVPGGRF
NCU04221.1	RPELRLDVQYLPEKITPEIVRDMNAKPGLLAVDMEEVVDP-KTGEKTLRGRPFVVPGGRF
FG09895.1 	RPEMRLDVQMLPEEITTDLVRDMNDAPGLLAVDVQEVSEPEHPSGWTLKGMPFVVPGGRF
AN5635.1  	KPELRLDVQELAAEITPEYVRDLNEKPGLLALAMEEKYDE-KTGKTDFAGVPFVVPGGRF
          	:*::****  *.  **.: * .:*  *****: ::*  :  :..   : * *********

MG09471.1 	NELYGWDSYMESLGLLVNDKVYLAKSMVLNFCFCIKHYGKILNATRSYYLCRSQPPFLTD
NCU04221.1	NELYGWDSYMESLGLLVNDRVDLAKSMVQNFCFCIKHYGKILNATRSYYLCRSQPPFLTD
FG09895.1 	NELYGWDSYMASLGLLINDRVDLAKSMVINFCFCIEHYGKILNATRSYYLCRSQPPFLTD
AN5635.1  	NELYGWDSYMESLGLLASNRVDLAKAMVINFCFCIKHYGKILNANRSYYLTRSQPPFLTD
          	********** ***** .::* ***:** ******:********.***** *********

MG09471.1 	MALRVYDKIRHEPDATEFLRTAILAAIKEYHSVWVAEPRLDPVTGLSRYRPEGTGVPPET
NCU04221.1	MTLRVYDKIKHEPGALEFLRQSLLAAIKEYYSVWTAEPRLDPVTGLSRYRPEGLGVPPET
FG09895.1 	MALRVYEKIKHEPDAKEFLRRSILAAIKEYHSVWVSEPRLDPTTGLSRYRPEGRGVPPET
AN5635.1  	MALRVYDRIQNEPGAMDFLRHAILAAIKEYYSVWMAEPRLDPVSGLSRYRSPGIGVPPET
          	*:****::*::**.* :*** ::*******:*** :******.:******. * ******

MG09471.1 	EADHFLHILEPYYKKHNMTFKEFVEAYNFGRIREPELDKYFLHDRAVRESGHDTSYRLEG
NCU04221.1	EAGHFIHILEPYAKKHNMSFDEFVYAYNHGEIKEPTLDDYFMHDRAVRESGHDTTYRFEG
FG09895.1 	EATHFVHILDPYIKKHNTTFEDFVRKYNHGEIEEPELDEYFMHDRAVRESGHDTSYRLEG
AN5635.1  	EASHFLHLLTPYAEKHGMEFKEFVQAYNYGKVKEPELDEYFMHDRAVRESGHDTSYRLER
          	** **:*:* ** :**.  *.:**  **.*.:.** **.**:************:**:* 

MG09471.1 	VCADLATVDLNTLLFKYETDIARTIRNVFGDKLVIPAEYCVG-SLQPGQVETSAIWDRRS
NCU04221.1	ICADLATIDLNSLLFKYETDIARTIRNVFHDKFEVPDDWLATNNPAASKLETSAMWDRRA
FG09895.1 	VCANLATIDLNSLLFKYETDIARTIRSVFNDKLTMPEEFCAGTPYQPGEVLSSAAWDRRA
AN5635.1  	VCGNLATVDLNSLLYKYEVDIARVIRVYFKDKLEIPVEFRTP-ATKDIQSESSSVWDRRA
          	:*.:***:***:**:***.****.**  * **: :* :: .       :  :*: ****:

MG09471.1 	KRRKLAIDKYLWNEEAGMYFDYDTAKRQQCNYESCTTFWALWAGVASPKQAAIMVTRALP
NCU04221.1	KRRKLAIDKYLWNEEAGMYFDYNTATRKQCNYESATTFWALWAGVSNPKQAAAMVTKALP
FG09895.1 	KRRKLTVDKLMWDEKEGMFFDYDTAKRERCTYESCTTLWALWAGIATPKQAAEMVRKALP
AN5635.1  	RRRKMRMDTYLWDEEKGMYFDYDTVKQERTNYESATTLWAMWAGLVTPRQASAMITKALP
          	:***: :*. :*:*: **:***:*..::: .***.**:**:***: .*:**: *: :***

MG09471.1 	KFEAYGGLLSGTEESRGQIGLDRPNRQWDYPYGWAPQQMLAWTGLYRYSFTEEAERLAYK
NCU04221.1	KLEAFGGLLSGTKESRGEIGLERPNRQWDYPYGWAPQQILAWTGLYRYGFNEEAERLAYK
FG09895.1 	KFEAYGGLVSGTEESRGAVGLDRPNRQWDYPYGWPPQQMLAWTGLIRYSFTEEAERIAYK
AN5635.1  	RFEEFGGIVSGTEESRGAVGLNRPTRQWDYPYGWAPQQMLAWTGFARYGYQEEAERLAYK
          	::* :**::***:**** :**:**.*********.***:*****: **.: *****:***

MG09471.1 	WLFMITKAFSDFNGVVVEKYDVTRPVDPHRVDAEYGNQGLGFKGVAKEG2FGWVNASYIY
NCU04221.1	WLFMITKAFVDFNGVVVEKYDVTRPIDPHRVDAEYGNQGLDFKGVAKEG2FGWVNASYVY
FG09895.1 	WLFMVTKAFVDFHGVVVEKYDVTRPADPHRVDAEYGNQGLGFRGVNKEG2FAWVNASYIY
AN5635.1  	WLYMITKAFVDFNGVVVEKYDVTRPIDPHRVDAEYGNQGVDFKGAPREG2FGWVNASYVY
          	**:*:**** **:************ *************:.*:*. :** *.******:*

MG09471.1 	GLQIINAHMRRALGTLTPYDTFIKALEDNRNRALSEMV--
NCU04221.1	GLQIVNAHMRRALGTLTPYETFMKAVEENRNKALSELV--
FG09895.1 	GLQIINAHMRRALGALTPYDTLMKAIEQNEEKTLAGLLSS
AN5635.1  	GLEMLNAHQRRALGAVTPWETYSKAVSAQGSDTVLENRSE
          	**:::*** *****::**::*  **:. : . ::    :.
```
